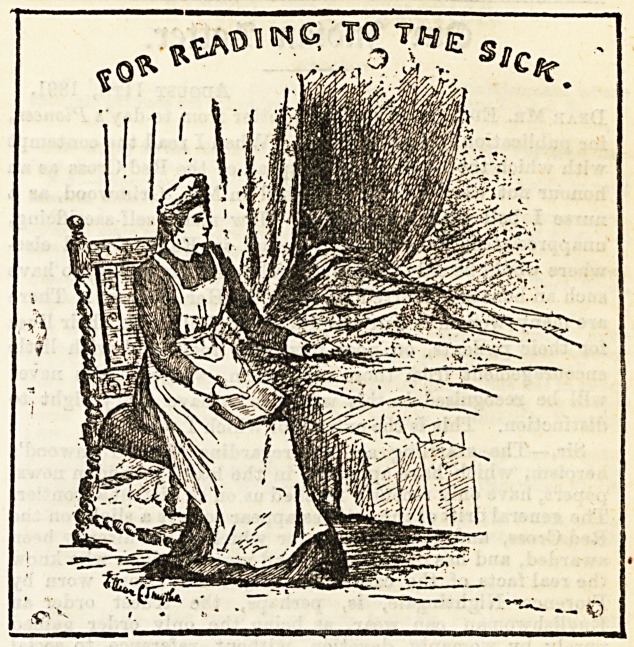# The Hospital Nursing Supplement

**Published:** 1891-09-19

**Authors:** 


### "The Hospital" Nursing Mirror.

Being the Extra Nursing Supplement of "The Hospital" Newspaper.

Contributions for this Supplement should be addressed to the Editor, The Hospital, 140, Strand, London, W.C., and should have the word "Nursing" plainly written in left-hand top corner of the envelope.

### En Passant.

RURAL NURSES.—The Rural Nursing Association has affiliated with the Queen Victoria Institute, and is now as "The Rural District Branch." This is a good move, especially as the Association has grown enormously lately; it has county centres for Devonshire, Hampshire, and Yorkshire, and 20 districts not under any local centre. Any clergyman wishing to have a district nurse in a rural parish should apply to Mrs. Malleson, for the fact that the nurses are "Queen's" nurses is a guarantee that they are properly qualified for the work they undertake.

UNSATISFACTORY NURSES. — A Superintendent sends us an account of two housemaids at hospitals who have been taken on at an institution as nurses. All we can do in this matter is to ask all medical men to be very particular not to take nurses from an agency which does not throw open its books to them, and show proofs of when and where their nurses were trained. With regard to these spurious nurses, and to the many drunken nurses going about, we are sorry that we cannot publish our black list, but we find that such a proceeding would be sure to land us in the law courts. However, any Matron who is dubious about a nurse applying to her can, by sending us a stamped addressed envelope, learn if the nurse is on our black list or not.

SHORT ITEMS.—We learn with pleasure that Messrs. Sampson Low, Marston, and Co. have severed their connection with the *Nursing Record*.—Miss Baxter, of the Children's Hospital, Cork, has had to take extra premises to house the numerous lady probationers crowding to the hospital.—The charges brought against the doctor and midwife of the Mount Street Dispensary at Belfast have been disproved.—Miss Conybeare, in writing to the *Woman's Herald*, from Cape Colony, states that Lady Lock is engaged in founding a Home for Nurses at Cape Town.—In our list of Queen's Nurses on September 5th, the name of Nurse Reader, of Worcester, was given as "Readee," by a printer's error.—Miss Clara Barton, of the American Red Cross Society, is the only woman who ever received the Iron Cross of Prussia from the old Kaiser William.

ANOTHER SOCIETY.—We hear that the society of operators in Massage and Medical Electricity is growing rapidly. The objects of the Society are to protect and develope the interests of operators in medical electricity and massage; to organise such operators (of both sexes) with a view to raising their calling into a distinct and recognized profession; to hold periodical meetings for discussion of subjects connected with the profession; to hold examinations (practical and theoretical), and award certificates; to publish a list of members (periodically) and send copies thereof to all hospital libraries, nursing institutions, and such clubs and societies as are frequented by doctors and nurses. We object to one point here, the granting of certificates. The Society will have to be very strong before it takes such a duty on itself, and in these early days it would have been better to be silent on the subject. Excellent work can be done in discussing the new methods which are constantly introduced, and in trying generally to combine and help masseurs and masseuses. But it is a pity to attempt too much, and to try to interfere with the privileges of the training schools.

MARRIAGE.—Miss Bettney, Matron of Stafford, will shortly be married to Mr. E. Teichelmann, F.R.C.S., and the two will depart for Australia. The engagement is an old one, and its consummation will secure congratulations from the Great Northern and Birmingham—both parties being well known in the latter town.

ALEXANDRIA.—On Tuesday, August 25th, His Highness the Khedive visited the Greek Hospital Alexandria, and was conducted over the building by Dr. Zancarol and the members of the Committee. The long corridors and wards were prettily decorated with Eastern plants and flowers. After inspecting the hospital, His Highness and suite adjourned to the sitting-room where coffee was served, and entered into conversation with the English sisters, the senior of whom, Miss Hamilton, wore the Khedivial Star of Merit, presented to her by the Khedive on the occasion of his visit two years before. Miss Aird was also present. His Highness expressed his satisfaction at all he saw before taking his leave.

NURSES' FOOD.—The *North British Mail* has taken up the cudgels in aid of the nurses and probationers of the Glasgow Royal Infirmary, who are said to work sixteen hours a-day, and to be badly fed. An instance is given of a probationer said to have been on duty for sixteen hours for nine consecutive days, and all the food, we are told, is miserably cooked and badly served. On Sundays the nurses have a cold dinner. "Why are the nurses not as well treated as the patients?" asks our Scotch contemporary. The answer is obvious; the nurses are or should be, healthy young women engaged in the honourable work of earning their own living, and to them a cold dinner should be satisfactory. The patients are ill and poor, and for the time being the recipients of charity; it is not well to compare their food with that of the nurses. It was quite time some mitigation was made in the labours of the Glasgow Royal nurses, but we could wish that they had had enough *esprit de corps* to bring their grievances before the Committee or medical staff, and had not washed their dirty linen in public.

HOUSEKEEPING DETAILS.—There is great wrath at Middlesborough because of the extraordinary disappearance of stock at the Fever Hospital. The Committee have just discovered a lack of linen and crockery that is truly deplorable; as Councillor Barron said, when they were shown over the hospital by Miss Going on her leaving, there was certainly plenty of crockery and towels, and the scales and weights were all right, but when they went up on Thursday there was only one two-pound weight for the scales, there were only three cups left, and there wasn't a towel for the nurses to dry their faces on. (Sensation.) Councillor Carter urged that the same system should be adopted in the Fever Hospital as in the Infirmary, where a proper inventory was kept by the Secretary of every article in the place, with columns in which to enter those destroyed or worn out. It is of course to this lack of an inventory and a regular period for "taking stock" that mischance is due. Every Matron should insist on an inventory and a quarterly inspection and correction of the same. The management of a large, or even of a small hospital, is a complicated business, and demands both method and training of the most efficient kind.

### A Fever Hospital.

(*By One Who Has Been There.*)

It often annoys me, now that I know better, to hear the slighting sort of way fever hospitals are sometimes spoken of. Now, I consider the only people whose opinion about them is worth having are those who have been patients in them, so in that case I consider my opinion about them worth having, for I was not only a patient but a trained nurse when admitted there. Now, how to get in as a patient is easy enough. As the Cookery-book says, "First catch your hare." Well, I say, first catch your fever; it is not a hard thing to do, especially if you are a private nurse. Get sent to nurse a case of scarlet fever when you are run down by a long run of bad cases, and you will not be many days before you are in agony with your throat; then comes the headache and sickness; out comes the rash, and you know your are in for it. Round comes the doctor to see his patient, and finds you also down. Then arises the question, "What are we to do with you?" They can hardly give you to the dustman, or the rag and bone man either; you know your own institute will not take you in, and you would not go to your own home even if you could; so, feeling as though life were well nigh over and nothing mattered, you murmur, "Send me to the fever hospital;" relief to the doctor's mind; he knew it was the only thing to be done, but did not like to propose it to you (yes, doctors have feelings, and do not regard nurses as the mere machines, as some people would have us believe); so off he goes, sends for an ambulance, which seems to arrive by magic, so quickly does it come, then up comes a nurse, you are divested of all your garments save one, enfolded in a huge white flannel garment like a monk's habit, and the porter is called up to carry you down. Luckily the porters are usually big men, for their burden is not always a little one! You fondly hope you will be carried down on a stretcher. No such thing; he picks you up and flings you over his shoulder like a dead sheep, and deposits you in the ambulance. Then you feel a sensation of comfort; a nice stretcher with an air cushion, soft pillows for your head, and warm blankets to cover you. The ambulance is well hung, and rolls you along swiftly and easily to the North-Western Fever Hospital. The gate porter opens the gates, and you are driven in, once more hung over the porter's shoulder, and carried into the receiving room. Then the receiving nurse takes possession of you; you are put into an easy-chair, rolled in blankets, and sit—feeling oh! so miserable—waiting for a doctor to come and admit you. It is rather a trying ordeal for a nurse to go through, for we are, perhaps, extra sensitive about some things, but there is not much time to think. In comes a doctor, examines your chest, tongue, throat, &c.; yes, there is no doubt you have it (you are only too painfully conscious of the fact). Then he asks you a few questions as to how you contracted it, which you answer as well as your poor throat will allow you to. Hearing you are a nurse he is very kind; picks out a nice ward to send you to. You are once more picked up, this time in a chair by two porters, carried across the grounds, and finally deposited in a ward. A kind-looking nurse comes forward to receive you, tells the porter which bed you are to have; you are picked up and laid down. Oh, bliss! at last you can rest. After a little your female curiosity makes you open your eyes and take a survey. The first thing strikes you is the spotless cleanliness of everything, and the bright, cheerful appearance of the ward: it is gay with flowers and plants; you even notice a long table tastefully arranged with ornaments of various sorts. Surely you must be dreaming! Ornaments and flowers in a fever hospital? Yes, it is a fact. It is not the dreary barn you pictured to yourself. The nurses take as much pride in making their wards look pretty as fashionable ladies do in making their drawing-rooms. Then you gaze round on your fellow-patients, and find that you are, indeed, among all sorts and conditions of men—or I should say women, as, of course, I was in a female ward. Well, what matter? The very roughest of them proved kind and was always ready to do a kind action for a patient worse than herself. Presently you are brought some beef-tea nicely served on a tray covered with a clean white cloth; you begin to feel happier, but a new fear arises; you hear that the doctor who admitted you was not the Medical Superintendent. You begin to wonder what he will be like. Needless worry, he comes and you are content; you need now have no more fears as to how you will be treated and taken care of, his bright cheerful manner reassures you at once, and you feel as happy as you can under the circumstances; his kindness and attention never flag, he takes as much interest in you when you are convalescent as he did when you were ill, and oh, greatest comfort of all, to a nurse who is ill you feel that in him you may have perfect confidence. And the Matron many a weary hour has her kindness helped me to pass, busy life as hers is. She always made time to stop and speak a few cheerful words when she came into the ward. Yes, I have passed many a happy hour in a fever hospital. Christmas Day I spent there. I had, to tell the truth, dreaded it, but it was really a happy day. There were pretty Christmas trees in all the wards, and abundance of good things for everybody. I cannot close this paper without speaking of the kindness I received from the nurses. They could not have done more for me had I been one of their staff instead of a complete stranger. I must say it was with regret that, after many weeks (for mine was a long illness), I said good-bye to the much-dreaded fever hospital, and I shall always have a warm feeling of gratitude towards the Medical Superintendent, the Matron, the Night Superintendent, the day and night nurses, and all who so kindly helped to make the time I was with them happy.

### The Princess of Wales and the Nurses.

September 15th is now past, but a good number of photographs promised have not yet been received. In consideration of the probably unavoidable nature of the delay in most cases, Miss Pritchard will receive photographs until September 26th, but no more cabinets can be accepted under any consideration. Should all fulfil their promise, the minimum of photographs required will be forthcoming. The prompt response which our appeal for a larger sum than could be thus collected by a subscription of 2s. will permit of a really pretty screen being provided, not unworthy of the Princess's acceptance. Subscriptions are acknowledged from the following nurses: C. Rodgers, M. A. Churchill, S. H. Stead, J. Flinley, E. Baylis, A. Wass, E. Harding, P. Coulson, A. Morton, E. Cowell, F. Barker, N. Thompson, E. Oxtoley, M. Walsham, A. Thomson, C Philp, N. Carter, E. Trott, A. James, E. Webb, H. Thompson, F. Dimsdale, Wright, A. Godwin, M. Macfarane, N. Lewis, S. Bates, E. Lowe, R. Lawrence, J. Buchan, Neuve, A. Bucktun, M. W., A. Sayers, E. Reid, M. Palmer, A. Holness, E. Moss, C. Meyer, E. Edge, Bradford, M. Underwood, S. Ward, M. Whitehead, S. Payne, J. Dinwoodie, M. Wilson, E. Turner, S. Lowers, E. Cavill, M. Rickarby, M. C. Lowghin, C. Robinson, B. Davies, King, M. Weller, A. Willson, A. Campbell, E. Wylie, E. Duggan, E. E. Jennings, Abel, A. Galbraith, F. Arnall, T. A. Smith, A. Parker, R. Frith, A. Lee, A. Dowling, E. Yeats, H. Gardiner, S. Assinder.

The following Matrons and nurses have responded in a hearty manner to our appeal for additional subscriptions towards the screen. S. Ginhum, 5s.; Galbraith, 3s.; M. A. W., 5s.; Evans, 5s.; A. Campbell, 3s.; A. Bates, 2s.; G. Medhurst, 2s.; M. Dinsdale, 5s.; E. Foster, 2s.; E. Norman, 5s.; E. Burton. 2s.; H. Newman, 3s.; C. McRae, 2s.; P. Daes, 1s.; E. Durham, 5s.; A. Fuller, 2s. 6d.; F. Elms, 3s.; A. Garratt, 3s.; H. Walker, 2s. 6d.; Denman, 2s.; Policy 1033, 1s.; Robinson, 1s.; Dormer, 1s.; J. Nelson, 3s.; E. Bishop, 2s. 6d.; L. Fisher, A. Waters, Ayrton, 3s.; A Nurse of over 25 years' standing, 5s.; A. Galbraith, 1s; E. Burgess, 3s.; M. Shipley, 3s.; M. Horton, 3s.; J. R. Dinwoodie, 3s.

No further list of acknowledgments will appear until after October 1st.

Errata.—Nurse Matthews should be read Matthens, and Nurse Pills should be Pitts, both in former lists.

A Nurse (photographer, Greaves, Halifax) has sent photograph and postal order without name or policy affixed.

### A Few Words to Nurses.

A THOROUGH nurse should have a strong sense of duty and a determination to obey all rules, whether they approve themselves to her or not. She should be always on the alert, but never in a hurry, doing everything with that brisk deliberation which is so assuring to both doctor and patient. She should be courteous to all about her and pitiful to those in her charge, grave without moroseness, cheerful without lightness, and have an unwearied love for her work.

That is a perfect woman, you say, and how can you, with all the toil and worry of a nursing life, hope to be like it?

We grant you it is a difficult task, and only she who gives of her best, who is willing to spend and be spent in Christ's service, who believes that in tending the suffering she is tending Him; that woman alone is likely to reach its perfection. We shall doubtless fall short of this pattern; yet after every fall we can just try again, and so "rise by stepping-stones of our dead selves to higher things."

Now to go into details, theoretically we are quite sure we intend to do our duty; but practically, duty has an uncomfortable knack of making itself disagreeable. For instance, we do not mind doing this or that particular thing, but—— or, this repulsive task would be easier if done at some other time, we think, only not just now; so we are naturally put out and lose our temper, and with it go patience and courtesy, and if the duty be done, it is not performed in the kindest or most gracious manner in the world. The best remedy for this is to have sympathy with our patients and to foster that grace as it is the out-come of the love which beareth all things and endureth all things. "A fellow feeling makes us wondrous kind," and nothing but sympathy will make us ready to "put ourselves in his place."

I remember a patient saying to a nurse who objected to perform an unpleasant duty for him, "It is hard enough, nurse, to have to ask you, to be dependent on you—you needn't make it harder."

If we could but let our imaginations have full play, and realise what we should feel if we were sick and suffering, dependent on strangers in a strange place, without a soul to whom we can unburthen our griefs, we should most probably substitute a gentler action, or a milder word than the one which rises to our lips. Rules there are which must be carried out, and we know also that patients are apt to consider they are made on purpose to annoy them, while food and medicine seem always administered at the wrong minute, still a gentle word, a kind look, a touch of sympathy, smooth away difficulties on both sides, the patient feels he has a friend with him, and the nurse that she has fought and conquered. Many a tough battle has come off by the bedside in a hospital ward.

Again, we can have sympathy with what may be termed the chill off, *i.e.*, an off-hand, neither hot nor cold manner. A nurse, herself the victim of a fell disease, told me once, 'When my patients used to say, 'Oh! nurse, I am so tired, I would reply, "Yes dear, I know, but," she went on, "Miss M., I did not know then, I do know now." Another who had worked some time with a fine sense both of duty and sympathy, but who has given up the profession, says that whenever she is in the least tired, she remembers with a pang every impatient word she has ever said to her patients and every failure to give them all the courtesy and care they needed.

We do not profess to do more here than point out failures and suggest helps to counteract them. For the use of those who have consecrated their lives to this especial following of Christ, there are many manuals and books of devotion, but to the ordinary lay nurse who does not feel this vocation we would say, spend a short time, if only a few minutes every morning in asking God's help on the coming labours of the day. If done earnestly, the habit of seeking Him at all moments of doubt and anxiety will grow. It is the attitude of the mind which is so often at fault. We cannot be always on our knees in a busy life, but *Laborare est orare* labour is a kind of prayer, when done in the fear of the Lord. Henceforth we will take St. Peter's counsel for our watchword, "Be pitiful, be courteous," and our Lord's tender love and sympathy with suffering for our Great Example.

### THE LESSONS WE TEACH.

WHILE we are very sick, we try to think what can have occasioned our malady, and how we may avoid a return of it when we get well. It is a wise precaution to take, because, except in the case of an accident, we may be pretty sure that our sickness arises from one of the following reasons—such as over-work; exposure to heat or cold, according to the season; improper food; or insufficient clothing. Now, most of these things can be remedied in a measure if we have a mind to try and alter them, so it is a good plan to give our past surroundings a thought. But, while we are doing it, let us remember that hearts and souls can be sick also, and we had better find out what we must avoid to restore them to health, for the mind and body act on each other, and when one is out of order the other follows suit. But we will only dwell on our faults long enough to find them out, and set to at once to put them to rights. One fault there is which besets us always in health, as well as in sickness—that is selfishness. How happy should we be if we could but forget ourselves and think more of those about us, but, unfortunately, in health we are ever upon the look-out for the best we can get; and in sickness we think the accident or the disease, whatever it may be, is sent to teach a lesson, especially to me. But may it not be a lesson for other people also? We may benefit others by bearing our sufferings and helplessness with patience and resignation; our cheerfulness will draw out the sympathy of our relations and friends; they will learn from us to "feel for others woes."

Do we ever think that we are not a solitary being, but part of a great body, just a member of that family which is in Christ? We know that if one limb or member of our body suffers all the others suffer likewise. For instance, if we break an arm or a leg, our head aches and we feel ill in every part; so if one person is wicked, or ill-tempered, or vindictive, other persons suffer for these faults. In like manner our patience, and resignation, and cheerfulness help our neighbours and friends to be happy. It is a very consoling thought that we are members one of another, and it helps us to feel less lonely when cut off from active life. We can pray for those that are in trouble, sorrow, need, sickness, or any other adversity, and feel that the prayers of others are going up to help us in our trials. Do not think we stand alone, and so have no influence. On the contrary, we can be teachers to those around us, and give by our example the best lessons in the world, even as our Lord Jesus showed by His life and death, the perfect pattern which we should copy.

### Our Indian Letter.

August 11th, 1891.

Dear Mr. Editor,—I enclose a letter from to-day's *Pioneer*, for publication in The Hospital. When I read the contempt with which the English papers speak of the Red Cross as an honour not worthy to be conferred on Mrs. Grimwood, as a nurse I boil with indignation. How many self-sacrificing, unappreciated, hard-working nurses in England and elsewhere would be only too proud, and would die happy, to have such an honour conferred on them by Her Majesty! There are many women in our hospitals who daily risk their lives for their patients, on poor pay, poor food, and with little encouragement from those over them, whose merits never will be recognised in this world, who have every right to distinction. This is the extract of which I spoke :—

Sir,—The startling articles regarding Mrs. Grimwood's heroism, which have appeared in the leading London newspapers, have only recently reached us on so distant a frontier. The general drift of the articles appear to cast a slight on the Red Cross, and the devotion for which it has already been awarded, and has disgusted beyond measure those who know the real facts of the case. The Royal Red Cross, worn by Florence Nightingale, is, perhaps, the truest order an Englishwoman can wear, as being the only order gained purely by womanly devotion without reference to social position. It was the fitting reward that her Sovereign thought fit to bestow on Mrs. Grimwood for her devotion in tending the wounded in the cellar during the truce, *not* under fire at any time, or while firing was going on, as has been stated, but still under very trying circumstances, which might well have accounted for a less heroic woman breaking down. Why then disparage the order by way of advancing Mrs. Grimwood's claims? In this devotion to the wounded lay her heroism; for this devotion she has been granted a special reward of £1,000, besides the Red Cross. The *Standard*, in discussing the affair, says, "certainly she tended the sick and wounded, but she would have been something less than an Englishwoman, if in that terrible time she had failed in so elementary a duty, so natural an impulse. But in the fierce attack on the Residency, when it would have been excusable for any woman to seek shelter, Mrs. Grimwood played the part of an active combatant." Her playing "the part of an active combatant," with "the service of a soldier and the example of a Joan of Arc" (*Daily Telegraph*) is an absolute fiction, like all the other sensational stories of her being wounded, her dressing the men's wounds, and tending the wounded under fire, together with the host of thrilling incidents attributed to the retreat, which the officers present utterly repudiate, and would like to know on whose authority these statements have been made. In conclusion, to speak of Mrs. Grimwood's decoration as "inadequate and unsuitable," "a comparatively commonplace honour" "a nurses' bow" is an insult both to the Order and to the heroine of Manipur. We enclose our cards. Truth.

I am going to institute myself your special correspondent here, for I see your readers are always asking questions about the Indian Nursing Service. Well, first, you must be over 25 years of age, be fully trained, and be ready to sign for five years' service if you wish to come to India. Then in return you get an allowance for an outfit, a free passage out, and 175 rupees per mensem from the day you sail. Then your name goes into the Indian Army List, and you hold a certain social position which is not to be despised. But the work out here is very heavy, the climate trying, and the discipline and red-tape irksome. I go on duty every morning at half-past five a.m., and work till half past eleven, then home for breakfast and a siesta; duty again from three till five p.m. or till eight p.m. every other day. The cases are mostly enteric, and have to be alternately sponged and fed all day and all night, and temperatures taken every four hours. One man's temperature was so high lately that it broke the glass, and we constantly chart up to 109°. That was during the heat; and, oh! read Rudyard Kipling if you want a description of the heat. I cannot give it. It recalls my sufferings now, when in the rains, I only want to forget the past.

The life is intensely interesting out here. The Sisters are stationed at Rawal-pundi, Lucknow, Meerut, and all sorts of pleasing places; but it would be hopeless save for the young and strong, so trying is the life. I hear that Mrs. Isa Foggo, Superintendent of the Zenana Hospital at Simla, is dead.

### Everybody's Opinion.

*[Correspondence on all subjects is invited, but we cannot in any way be responsible for the opinions expressed by our correspondents. No communications can be entertained if the name and address of the correspondent is not given, or unless one side of the paper only be written on.]*

### DRUNKEN NURSES.

Miss Mattocks writes: I read with great pleasure in your last week's number the very sensible letter of "A Superintendent" anent nurses' food, and I also read with shame and sorrow of how the "Superintendent of a Nurses' Home" had been afflicted by receiving a "nurse" whose degraded state made her a libel on her profession; and I believe that the evils mentioned by the writers may be classified as cause and effect in many cases. When nurses, after a hard morning's work, come down day after day to dinners whose only variety consists in the different ways they have been spoilt in cooking, it cannot be surprising that people with work to do that needs all their mental and physical strength to perform should be tempted to seek from stimulants the energy they are unable to obtain from untasted dinners and tea and bread and butter. It is usually the women who are past their youth and have neither the appetite nor digestion of early years who fall victims to the insidious temptation which ends in their ruin. Yet it is not the want of means, but of heart and thought which leads to such mischief. A varied diet costs no more—except in brains—than a monotonous one, though its value to health and pleasure may be treble. I fear, however, that there will be little improvement in these matters whilst Matrons are chosen solely for their efficiency in the work and management of hospital wards without any guarantee as to their knowledge of domestic management, which certainly cannot be acquired there.

### THE R.B.N.A.

Miss K. M. Heanley, Boston Hospital, Lincolnshire, writes: Will you again admit a letter from me re *Nursing Record*, as the editor does not do me the justice to publish my letter, while he admits many written against my action. I have shown no disloyalty to the Association, but I and others with me object to the tone of the *Nursing Record*, which styles itself "The organ of the Nursing Profession." We object to being so misrepresented. I, and other members of the R.B.N.A., object to such partizanship as that shown for us by the *Nursing Record*. I further wish to call attention to the fact that in a foot note the *Nursing Record* states "it has not, and never has had, any connection with the R.B.N.A.," thus justifying my desire that the R.B.N.A. on its side should assert the same.

### OUT-DOOR UNIFORM.

"A Nurse for Seven Years" writes: I am somewhat surprised to find one of the nursing world so against out-door uniform. Nurses, private especially, frequently have only time enough for their walk without having to change their dresses. We seldom find patients object, in some cases it is preferred, and, as to attracting attention, a nurse must be taking more notice than is necessary, to become conscious that she is attracting so much attention, as there are many professions which render it necessary and sometimes compulsory to wear out-door uniform. In the case of going to celebration a dirty apron is not nice, but the nurse is surely to be commended for thinking more of the blessed Sacrament than her personal appearance; if the apron is septic through being worn under a cloak, why not the dress also? We admit there are many ugly uniforms, but fail to see why a plain neat uniform can be objected to; in fact, we think it much better than the ordinary cloak or ulster, or even dresses put over indoor uniform, which so many nurses are in the habit of doing.

### CORRECTIONS.

A CORRESPONDENT writes: May I draw your attention to a mistake made by the author of "How Doctors are Made," in this week's HOSPITAL? He states: "In order to take a degree in medicine at Cambridge, a man must take his degree in Arts, his B.A." The distinguishing point between Oxford and Cambridge medical degrees is the fact that at Oxford this is necessary, and is not so in the case of Cambridge. This distinction is claimed by Oxford, rightly or wrongly, as enhancing the value of the Oxford degree.

THE LADY SUPERINTENDENT, Nurses' Institute, Canterbury, writes: Will you allow me space in your columns to correct two mistakes in your paragraph last week referring to my resignation, which you state "was sent in on account of the action taken by the Ladies' Committee in respect to the District Nurse, concerning whom unpleasant rumours were afloat." In the first place, my resignation was sent in on purely personal grounds, and has nothing whatever to do with that of the District Nurse. In the second place, the words "unpleasant rumours" are totally unfounded, and out of place as connected with the said District Nurse. A want of the necessary support and sympathy from many of the medical men and others in Canterbury, creating a dearth of cases, caused her, as it would anyone having a true love of their work, to resign her post here, which resignation was accepted, the sick poor thereby losing a nurse whose devotion to and skilful management of her cases will not soon be forgotten by those who have reason to be grateful to her, and one whom the Committee will find it difficult to replace. [We regret the publication of this letter has been delayed.—EDITOR.]

### Appointments.

[It is requested that successful candidates will send a copy of their applications and testimonials, with date of election, to THE EDITOR, The Lodge, Porchester Square, W.]

BOLTON BOROUGH HOSPITAL.—Miss Mariquita Clark has been chosen Matron of this hospital out of 19 applicants. Miss Clark trained at the Northern Hospital, Liverpool, and was afterwards for three years at the Royal United Hospital, Bath. Latterly Miss Clark has acted as Night Superintendent at the Bolton Infirmary, and has there won excellent testimonials.

CHILDREN'S ORTHOPÆDIC HOSPITAL.—Miss Shelley has been appointed Matron of this Dublin Hospital, in place of Miss Macintosh, who is leaving to be married.

GLAMORGAN AND MONMOUTHSHIRE INFIRMARY.—Miss L. A. Mont Wilson has been appointed Matron of this Infirmary at Cardiff. Miss Wilson trained at the Adelaide Hospital, Dublin; then went to Maidstone, where she spent many years; latterly she has acted as Matron at Tewkesbury and the Highgate Children's Hospital.

### Presentation.

BOLTON INFIRMARY.—Sister Mariquita, on resigning the post of Night Superintendent, was presented by the Matron, Sisters, and Nurses with a very chaste afternoon tea service complete, as a mark of their esteem. Also from one of the Nurses with a Russian leather cardcase with silver mountings.

### Notes and Queries.

### Queries.

(47) Can any of your readers tell me of an establishment where epileptic patients are taken for a small weekly payment?—*A.C.S.*

### Answers.

(41) Apply to the Hospital for Epilepsy and Paralysis, Portland Terrace, Regent's Park, N.W. Charge from 10s. 6d. a-week.

*Bob.*—Superfluous hairs can be removed by electricity. Apply to your medical attendant.

*A. B. J. T.*—We know of no institute or hospital which grants certificates to male nurses, save certain schools of massage. Your American certificates will be recognised here.

(46) Apply to Sister E., 132, Christchurch, Boscombe (a guinea a week), or to Miss Spinks, 1, Crowther Terrace, Upper Torr Road, Westcliff, Bournemouth.

*X. Y. Z.*—We do not give medical advice, or admit medical queries.

*A Would-be Nurse.*—You had better get "The Hospital Annual," price 3s. 6d., from this office, or look up our back numbers. Your queries have been constantly answered before. A probationer is generally on duty twelve hours a day, and has two hours off duty. She has to sweep and dust the ward, make the beds, and do as she is told by the charge nurse. Get "Sketches of Hospital Life," price 1s., published by Sampson Low.

*Cremation.*—Apply to the Secretary, Necropolis Company, 2, Lancaster Place Strand. This for practical purposes. The Hon. Secretary of the Cremation Society, is Mr. Swinburne-Hanham, 8, New Cavendish Street, London, W.

*Rhoda.*—Do you dip your hands into too strong carbolic? If so, that is probably the cause of the roughness. You can rub on vaseline at night and wear gloves, but after all—

"Beautiful hands are those that do,

Work which is honest, and brave, and true,

Moment by moment the whole day through."

*Nurse Bayes.*—We know of no such institute or bazaar; as a rule nurses have no time for other work save tending the sick.

*Sister Winifred.*—Write to the Secretary, Royal National Pension Fund for Nurses, 8, King Street, Cheapside, E.C.

(42) Get "Antiseptics," by Mrs. Hewer. Published by Crosby Lockwood, price 1s.

(43) Certainly it is safer to filter drinking water through a good charcoal filter.

*Heathfield.*—Mittens received with thanks.

### Wants and Workers.

*Acknowledgments.*—The Lady Superintendent of King's Lynn thanks C. S. Blackheath, Miss Hertz, Anon, and P. O., Portland Street, for their help.

*Old Linen Wanted.*—The District Nurse, 82, Victoria Street, Ashton-under-Lyne, appeals for old linen and underclothing, and some large stockings, to help with bad cancer cases, and some cases of bad legs.

### Amusements and Relaxation.

### SPECIAL NOTICE TO CORRESPONDENTS.

**Third Quarterly Word Competition, commenced July 4th, 1891, ends September 26th, 1891.**

**Competitors can enter for *all* quarterly competitions, but no competitor can take more than *one first prize* or *two* prizes of any kind during the year.**

Proper names, abbreviations, foreign words, words of less than four letters, and repetitions are barred; plurals, and past and present participles of verbs, are allowed. Nuttall's *Standard* dictionary only to be used.

The word for dissection for this, the TWELFTH week of the quarter, being

"OLIVES."

| Names. | Sept. 10th. | Totals. | Names. | Sept. 10th. | Totals. |
|---|---|---|---|---|---|
| Paignton | 147 | 344 | Christie | — | 44 |
| Psyche | 165 | 465 | Dulcamara | 152 | 439 |
| Hope | — | 47 | Nurse J. S. | 147 | 439 |
| Lightowlers | 164 | 478 | Qu'appelle | 171 | 429 |
| Wizard | — | 179 | E. M. S. | — | 68 |
| Wyameris | — | 46 | Jenny Wren | 151 | 434 |
| Dove | — | 46 | Carpe-diem | — | 65 |
| Punch | — | 181 | Grannie | — | 36 |
| Ivanhoe | — | — | Nurse G. P. | 100 | 272 |
| Tinie | — | 93 | Goodnight | — | 122 |
| Agamemnon | — | — | Gamp | — | 100 |
| Nurse Ellen | — | 86 | Charity | — | 104 |

### Notice to Correspondents.

**Fourth Quarterly Word Competition commences October 3rd, 1891.**

All letters referring to this page which do not arrive at **140, Strand, London, W.C.**, by the *first post on Thursdays*, and are not addressed PRIZE EDITOR, will in future be disqualified and disregarded.

N.B.—Each paper must be signed by the author with his or her real name and address. A *nom de plume* may be added if the writer does not desire to be referred to by us by his real name. In the case of all prize-winners, however, the real name and address will be published

### Content to Follow.

Oh righteous doom! that they who make
Pleasure their only end,
Ordering their whole life for its sake,
Lose that whereto they tend.
While they who bid stern duty lead,
Content to follow—they
Of duty only taking heed,
Find pleasure by the way.—*R. C. Trench.*

The following story of a woman's devotion to duty and cheerful sacrifice of self, took place many years ago before sick nursing among the poor had become general, as it is now.

Indeed, in those days, it hardly existed except in instances, which were the exception and not the rule.

It was at that time also quite the exception for any well-educated refined woman to take up sick nursing as a vocation.

Mary Rayson had gone through a course of training, first in a German "Krankenhaus," and then in one of the large London hospitals, a training far harder in every detail than any nurse has experience of in these days, when so many of the rough stones and sharp thorns, which formerly made the career a hard one, have been removed from the nurse's path.

She had now come to enjoy a well-earned rest for a few weeks at the house of a friend in a small country village. She had lately become engaged to a doctor with whom she had worked, and who, having obtained a good appointment in India, had just sailed, full of happy expectation that the day was not very far distant when Mary would go out and make his home all that a home can be.

But happy dreams of the future did not prevent her from taking a very practical interest in the present. She had not been many days in the village before she heard of several cases of low fever among the poor people, and with true womanly instinct to help the sick, she gave up many hours of the day to what was to her truly a labour of love.

One day—late in the afternoon—she was returning home, and to save time passed down one of the smallest and poorest streets in the place.

An excited crowd had gathered round the door of one of these wretched-looking houses, and completely blocked the way.

She had turned to a woman standing near to inquire the cause, when out of the door strode a young man, who, with the air of one in authority, waved to the knot of people to stand back.

"Go away, all of you," he said, sharply; "no use to loaf about here, when not one of you will lift a finger to help him. A nice set you are! Ah, Miss Rayson is that you?" He came quickly over to where she was standing. "Not the place for you, though. You had better come on, and I will go back in a minute and see what can be done; you never saw such a set of frightened idiots in your life."

"What is it?" said Mary, who had no idea what he was talking about.

"Poor fellow up there," he said, waving his hand towards the house he had just left, "as bad as he can be. Dying, I expect, and not a creature in the village will go near him. There I found him more dead than alive. I cleared everyone out of the house when I saw what it was, and then asked for someone in common charity to attend to him, but not a creature will cross the doorstep now they have heard what it is. What can I do? My hands are full already. I ought to be at a case five miles off at the present moment, or I would stay with him."

"And what is the disease?"

"Oh, smallpox, unquestionably; of the worst type. That's what they are all so afraid of."

"Is there no place you could take him to?"

"No hospital for thirty miles, and it would kill him to try to move him. He must stay where he is; but it is horrible to think he will have to die alone. I only want one person; in fact, I would not allow anyone else to go into the house, but to think there is not one person in this place who will take the risk!"

"*I* will, Dr. Coles," she said quietly.

While he had been speaking she had seen, as in a vision, two ways lying before her. One, the path of discomfort and danger, to help a stranger who had no claim on her but the claim of common humanity; the other a path of apparent safety and ease. No one would blame her for choosing the latter; indeed, she knew well that had George, her lover, been there he would not have allowed her to go, but he was not there, and she knew well, too, that if she did not go, no one else would come forward to take the post of danger which she had shrunk from accepting.

It was not a question of inclination; it was a question of duty.

She saw all this quite clearly; and had not hesitated.

"Do ye nexte thinge" had been a favourite motto of hers; and now this duty had come to her through none of her own seeking or asking, she did not falter in her choice.

She "bade stern duty lead—content to follow" wherever it might take her.

Even while she was speaking she felt that valuable time was passing, and that her help was sorely wanted at the sick man's bedside.

"You will!" exclaimed the young Doctor, hardly believing his ears. "Ah! I had forgotten you were a trained nurse; but"—he paused—"you know what the risk is? It is one of the worst cases I have ever seen. No hope, I am afraid."

He did not guess the extent of the sacrifice that Mary had so quickly and quietly made, and was (after relieving his conscience by impressing the risk on her) only too glad to have such valuable help in his difficulty.

"I will go at once," she replied, "and send up to the Rectory for the things I shall want," clearly foreseeing that if she went she would probably meet with strong opposition to her plan, if not actually prevented by her friends from carrying it out.

"I will be back with you directly," Dr. Coles said. "I will send up disinfectants and everything you will want from my surgery. You will find very bare quarters there," and he was off.

On his return soon afterwards he found Mary quietly moving about the wretched room, trying to introduce some small degree of order at least, if not of comfort, into the squalid surroundings.

"Have you everything you want now?" he said. "My boy can bring anything you want, and leave it downstairs."

"A candle," suggested Mary with a smile, preparing with a brave heart for the weary vigil before her.

"Of course, how stupid of me, you would have been in the dark all night. I must go to a patient in the country at once, but I will be here as early as possible in the morning. Good night. How kind this is of you. I can never thank you enough."

And, indeed, he never knew how much he had to thank her for. What bright hopes and fair visions of the future were laid on one side that she might give her whole attention to the work, which had called her so unmistakably to this place. The call to do the duty had come to her, and to no one else, and she knew that she—and no one else—must turn and do this thing.

(*To be continued.*)

## Figures and Tables

**Figure f1:**